# Investigating the impact of sleep quality on cognitive functions among students in Tokyo, Japan, and London, UK

**DOI:** 10.3389/frsle.2025.1537997

**Published:** 2025-05-26

**Authors:** Joshua Ampofo, Binghai Sun, Geoffrey Bentum-Micah, Li Qinggong, Wang Changfeng, Le Guoan, Qian Xusheng

**Affiliations:** ^1^School of Psychology, Zhejiang Normal University, Jinhua, China; ^2^School of Public and Administration, University of Electronic Science and Technology of China, Chengdu, China; ^3^Centre for West African Studies, University of Electronic Science and Technology of China, Chengdu, China; ^4^College of Education, Zhejiang Normal University, Jinhua, China

**Keywords:** sleep quality, cognitive functions, university students, cross-sectional study, cultural differences

## Abstract

**Background:**

This study focuses on cultural influences and investigates sleep quality's impact on cognitive functions among university students in Tokyo and London. Recognizing sleep as vital for wellbeing and academic success, it explores factors affecting sleep quality and its cognitive impact in diverse educational settings.

**Methods:**

A cross-sectional study was conducted with 400 students (200 per city). The Pittsburgh Sleep Quality Index (PSQI) assesses sleep quality. At the same time, cognitive tests, including the Rey Auditory Verbal Learning Test and the Stroop Test, evaluated memory, attention, problem-solving, and executive functions.

**Results:**

Significant negative correlations emerged between PSQI scores and cognitive performance, indicating that poorer sleep quality correlated with diminished cognitive abilities across domains. Regression analyses confirmed sleep quality's predictive role in mental performance, controlling for demographics. These findings highlight sleep's critical role in cognitive functions within different cultural contexts.

**Conclusion:**

This study underscores sleep quality's importance for academic success and reveals cultural variations influencing sleep patterns among Tokyo and London students. The findings suggest targeted interventions to enhance sleep quality and cognitive functioning in diverse educational settings, considering cultural nuances and lifestyle factors. Further research should explore longitudinal effects and intervention strategies to improve sleep and mental outcomes.

## Introduction

The intricate relationship between sleep quality and cognitive functions has become a focal point in contemporary health research. It is increasingly recognized for its profound impact on overall wellbeing, particularly among university students. Sleep quality, defined by a combination of factors such as sleep duration, sleep continuity, and subjective sleep satisfaction, is essential for various cognitive processes (Bailey et al., 2023). Deficits in sleep quality have been consistently linked to impairments in key cognitive domains, including attention, memory, and executive functions, which are all critical for academic success and daily functioning. Moreover, recent research has expanded the scope of sleep quality's influence, highlighting its association with life satisfaction, mental health, and even congenital diseases (Lawson et al., 2020). These findings underscore the need for a deeper understanding of the factors that influence sleep quality and its subsequent impact on cognitive performance. Indeed, from an economic perspective, the implications of sleep disorders are vast, encompassing healthcare costs, productivity losses, and broader societal burdens (Runze et al., 2024).

The direct correlation between improved sleep quality and enhanced academic performance is well-documented. Adequate sleep is vital for memory consolidation and cognitive processing, as the brain actively processes and stabilizes new information during the rapid eye movement (REM) and slow-wave sleep (SWS) phases (Hoedlmoser et al., 2022). Insufficient sleep, on the other hand, can lead to considerable declines in cognitive abilities, affecting not only academic achievements but also broader aspects of life, such as emotional regulation and social interactions (MacCann et al., 2020). For example, chronic sleep deprivation has been shown to increase irritability, impair emotional stability, and diminish the capacity for empathetic social engagement (Simon et al., 2020). These effects emphasize the crucial role of sleep in fostering a balanced and healthy lifestyle. The neurological impacts of chronic sleep loss are particularly concerning, with evidence suggesting that prolonged sleep deficits can alter brain structure and function, particularly in areas responsible for stress response and emotional processing.

In light of the increasing academic demands and pressures faced by students today, it is imperative to investigate the relationship between sleep quality and cognitive functions within diverse educational and cultural contexts. This study focuses on university students in Tokyo, Japan, and London, UK, two metropolitan centers characterized by distinct cultural norms, academic environments, and societal expectations. Tokyo, known for its rigorous educational system and high societal standards, presents a unique environment in which academic achievement is often prioritized over personal wellbeing. This cultural emphasis on success can lead to chronic academic stress, inconsistent sleep patterns, and compromised sleep quality among students. The phenomenon of “presenteeism” in Japanese academic culture, where students are expected to be physically present for extended hours regardless of their actual productivity or health status, further exacerbates the issue (Nath et al., 2024).

In contrast, London offers a more diverse student population, encompassing varied lifestyles, cultural backgrounds, and approaches to managing academic stress. This diversity provides a valuable opportunity to examine how different cultural influences and lifestyle choices impact sleep quality and cognitive outcomes. While London's educational framework may offer more flexibility and encourage a better work-life balance, students in this context also face significant challenges related to academic pressure, financial strain, and access to resources that promote healthy sleep habits. Socioeconomic disparities can particularly affect students' ability to secure safe, quiet, and comfortable living environments conducive to restful sleep (Billings et al., 2020). Therefore, a comparative analysis of the relationship between sleep quality and cognitive functions in Tokyo and London is essential for developing targeted interventions that address the unique challenges faced by students in these distinct cultural contexts.

## Literature review

The complex interplay between sleep quality and cognitive functions has become a prominent area of research, particularly concerning university students. To fully understand this dynamic in the distinct settings of Tokyo, Japan, and London, UK, it is crucial to explore several interconnected theoretical frameworks: the Stress-Performance Relationship, the relationship between stress and mental health, and the impact of sleep quality on cognitive functions and mental health.

### Stress-performance relationship

The Stress-Performance Relationship posits that academic stress can significantly impair sleep quality, resulting in a cyclical pattern of sleep deprivation, increased stress, and diminished cognitive abilities (Gilbert et al., 2024). High academic demands, intense competition, and constant pressure to succeed often lead to anxiety and worry among students, disrupting normal sleep patterns and increasing the risk of insomnia. Studies have consistently shown that students reporting elevated stress levels also experience poorer sleep quality and heightened insomnia symptoms (Candia et al., 2023). This relationship is particularly relevant in the cultural contexts of Tokyo and London, where unique stressors can exacerbate sleep-related issues.

In Tokyo, the stringent academic environment and high societal expectations place immense pressure on students, contributing to chronic stress and anxiety. The cultural emphasis on achievement and conformity can create a climate of relentless competition, leading to sleep deprivation and burnout. A recent study by Wang and Matsuda (2023) found that Japanese university students who perceived high levels of academic stress were more likely to experience sleep disturbances and cognitive impairments. This is compounded by the “presenteeism” culture, where students feel compelled to attend classes and study for long hours, regardless of their physical or mental wellbeing (Kinman and Wray, 2022).

While offering a more diverse and flexible academic environment, London also presents unique student stressors. Financial pressures, the high cost of living, and concerns about future career prospects can contribute to significant stress and anxiety. A study by Tang et al. (2022) revealed that London University students who reported financial difficulties were more likely to experience sleep disturbances and mental health issues. Moreover, the multicultural nature of London can create additional challenges for students from diverse backgrounds, who may experience acculturative stress and social isolation, further impacting their sleep quality.

### Stress and mental health

The relationship between stress and mental health is well-established, with chronic stress serving as a significant risk factor for various mental health disorders, including anxiety, depression, and burnout (Pascoe et al., 2020). Elevated stress levels can disrupt the delicate balance of neurotransmitters in the brain, leading to mood disturbances, cognitive impairments, and emotional dysregulation. Students who experience chronic stress are more vulnerable to developing mental health problems, which can further exacerbate sleep disturbances and cognitive deficits.

In Tokyo, the intense academic pressure and cultural stigma surrounding mental health issues can create a perfect storm for students at risk. The reluctance to seek help for mental health concerns can lead to untreated conditions, further compromising sleep quality and cognitive function. A survey by Ishida et al. (2020) found that Japanese university students with high levels of stress were less likely to seek mental health support despite reporting symptoms of anxiety and depression.

London, while having more accessible mental health resources, still faces challenges related to stigma, awareness, and accessibility. The high demand for mental health services can lead to long waiting lists, making it difficult for students to access timely and effective support. A study by Coelho et al. (2022) found that London University students from low-income backgrounds were less likely to receive mental health treatment due to financial barriers and lack of awareness. Addressing students' mental health needs is essential for improving sleep quality and promoting cognitive wellbeing. Interventions such as stress management workshops, counseling services, and mental health awareness campaigns can help reduce the burden of stress and improve mental health outcomes among students in Tokyo and London.

### Sleep quality and cognitive functions (mental health)

The impact of sleep quality on cognitive functions has been extensively documented, with studies consistently demonstrating that poor sleep quality impairs attention, memory, executive functions, and overall cognitive performance (Leong and Chee, 2023). Sleep is crucial for memory consolidation, restoration, and regulating neurotransmitter systems supporting cognitive processes. Chronic sleep deprivation can lead to structural and functional changes in the brain, affecting cognitive abilities and increasing the risk of mental health disorders (Killgore et al., 2022).

In Tokyo, where long study hours and irregular sleep schedules are common, the impact of sleep deprivation on cognitive functions is particularly concerning. Japanese students who sacrifice sleep to meet academic demands often experience impaired attention, reduced memory capacity, and diminished problem-solving skills. Hicks et al. (2023) found that Japanese university students with poor sleep quality performed significantly worse on cognitive tests measuring attention, memory, and executive functions.

London students also face challenges related to sleep quality and cognitive functions. Lifestyle factors such as social jetlag, exposure to electronic devices before bed, and irregular work schedules can disrupt sleep patterns and impair cognitive performance. Li et al. (2024) found that London University students who frequently used smartphones and tablets before bed reported poorer sleep quality and reduced cognitive abilities. Moreover, the high levels of stress and anxiety experienced by London students can further compromise sleep quality, leading to a vicious cycle of sleep deprivation and cognitive impairment.

Importantly, there is a bidirectional relationship between sleep quality and mental health. Poor sleep quality can exacerbate mental health symptoms, while mental health disorders can disrupt sleep patterns. This interplay highlights the need for interventions that simultaneously address both sleep and mental health issues. Cognitive-behavioral therapy for insomnia (CBT-I) is effective in improving sleep quality and reducing symptoms of anxiety and depression (Benz et al., 2020). Mindfulness-based interventions have also been found to promote relaxation, reduce stress, and improve student sleep quality. The Stress-Performance Relationship, stress and mental health, and the impact of sleep quality on cognitive functions and mental health are intricately linked and highly relevant to the proposed study in Tokyo and London. By examining these relationships within these two cities' specific cultural and academic contexts, this research aims to provide a comprehensive understanding of the factors that influence sleep quality and cognitive wellbeing among university students.

## Theoretical framework

This study draws upon several key theoretical frameworks: memory Consolidation Theory, Cognitive Load Theory, Circadian Rhythm Theory, and the Stress-Performance Relationship. These frameworks offer distinct but interconnected perspectives on how sleep influences cognitive processes and are essential for interpreting the findings of this research.

### Memory consolidation theory

The Memory Consolidation Theory posits that sleep is crucial for consolidating memories formed during wakefulness. Specifically, during sleep phases like REM and slow-wave sleep (SWS), the brain actively processes and stabilizes newly acquired information, effectively transferring it from short-term to long-term memory stores (Sridhar et al., 2023). Disruptions in sleep patterns, therefore, can significantly impair this consolidation process, leading to deficits in both declarative memory (facts and events) and procedural memory (skills and tasks) (Menzies et al., 2025). This directly impacts academic performance, as students require efficient memory consolidation to retain and recall information effectively.

In the cultural context of Tokyo, where long study hours are often prioritized oversleep, the implications of disrupted memory consolidation are particularly salient. Students may sacrifice sleep to meet academic demands, inadvertently hindering their ability to retain the information they are studying. The cultural emphasis on academic achievement and the demanding curriculum can lead to a cycle of sleep deprivation and impaired memory consolidation, negatively impacting academic outcomes. The pressure to excel can override the understanding of sleep as a necessary learning component. This context reinforces the significance of examining how sleep patterns impact memory consolidation among students in Tokyo.

In contrast, London's more diverse and potentially less rigid academic environment may present different challenges. While there might be less overt pressure to study long hours, students in London can still experience disruptions in memory consolidation due to stress, irregular sleep schedules, and lifestyle factors. The diverse cultural backgrounds of students in London may also lead to variations in sleep habits and their impact on memory consolidation. The cultural context in London calls for an understanding of how diverse lifestyles affect sleep patterns and, thus, memory consolidation. This theory provides a crucial lens through which to examine the impact of sleep quality on memory retention and recall among students in Tokyo and London. By assessing and correlating memory performance with sleep quality, this study can determine whether cultural differences in sleep habits mediate the relationship between sleep and memory consolidation.

### Cognitive load theory

Cognitive Load Theory highlights working memory capacity limitations and the importance of minimizing cognitive load to optimize learning and performance. The theory defines cognitive load as the mental effort to process information in working memory. When students experience subpar sleep quality, their cognitive resources become strained, and the cognitive demands of academic tasks can become overwhelming (Chew and Cerbin, 2021). This strain can hinder attention, executive functions, and overall information processing efficiency, ultimately diminishing academic performance.

Cultural and environmental factors can influence cognitive load and its relationship with sleep quality. In Tokyo, the intense academic environment, demanding curriculum, and competitive nature of the educational system can impose a high cognitive load on students. This high cognitive load, combined with sleep deprivation, can create a detrimental cycle of cognitive overload and impaired learning. Japanese students may struggle to process and retain information effectively due to the combined effects of high cognitive demands and insufficient sleep.

While the academic environment in London may be less structured, students face different sources of cognitive load. These include financial pressures, social challenges, and adapting to a diverse and multicultural environment. Students from disadvantaged backgrounds may experience higher levels of stress and cognitive fatigue due to financial constraints and limited access to resources. The interplay between these stressors and sleep quality can significantly impact London students' cognitive load and academic performance. This theory helps frame the study by emphasizing the limited cognitive resources available to students and how lack of sleep can severely impair that capacity. This study can explore how various dimensions of sleep quality relate to cognitive load among students in Tokyo and London, considering the cultural and environmental factors that contribute to cognitive strain.

### Circadian rhythm theory

The Circadian Rhythm Theory focuses on the importance of biological clocks in regulating sleep-wake cycles and their impact on cognitive performance. Circadian rhythms govern numerous physiological processes, including hormone release, body temperature, and alertness, which fluctuate throughout the day. Disruptions in circadian rhythms, often caused by irregular sleep patterns or environmental influences, can lead to a misalignment between optimal cognitive performance periods and academic demands (Heller et al., 2024). This misalignment can negatively affect attention, memory, and executive functions, impairing academic performance.

In Tokyo, the demanding academic schedule and cultural norms prioritizing productivity over rest can disrupt students' natural circadian rhythms. Many students engage in late-night study sessions and have early morning classes, leading to chronic sleep deprivation and circadian misalignment. The reliance on technology and artificial light, particularly in densely populated urban areas, can further disrupt circadian rhythms, exacerbating sleep-related cognitive impairments.

London students may also experience circadian disruptions due to social jetlag (the discrepancy between weekday and weekend sleep schedules), exposure to electronic devices before bed, and shift work. The vibrant nightlife in London and the prevalence of irregular work schedules can disrupt sleep patterns and desynchronize circadian rhythms. These disruptions can negatively impact cognitive function and academic performance, particularly for students who naturally prefer later sleep-wake cycles. This theory is crucial because it accounts for specific sleeping patterns and preferences. Understanding how sleep patterns impact health and academic performance is key for educational entities. This study can investigate how circadian alignment relates to sleep quality and cognitive performance among students in Tokyo and London, considering cultural and environmental factors that influence sleep-wake cycles.

These theoretical frameworks provide a comprehensive lens to understand the complex interplay between sleep quality, cognitive functions, and cultural contexts in Tokyo and London. Memory Consolidation Theory highlights the importance of sleep for memory retention. Cognitive Load Theory emphasizes the importance of minimizing cognitive demands during sleep deprivation. Circadian Rhythm Theory underscores the significance of aligning sleep-wake cycles with natural biological rhythms, and the Stress-Performance Relationship illuminates the cyclical relationship between stress and sleep. By applying these frameworks, this study provided valuable insights into the specific challenges faced by students in Tokyo and London. The findings informed the development of targeted interventions to improve sleep quality, reduce stress, and enhance cognitive function among university students. This research can potentially contribute to educational policies and practices prioritizing student wellbeing and promoting academic success.

### Research gaps

While existing literature has significantly advanced our understanding of the relationship between sleep quality and cognitive outcomes, notable gaps remain. Many studies have primarily focused on Western populations or older adults, resulting in a lack of data on young adults in non-Western societies. The absence of diversity in research samples limits our understanding of how cultural contexts influence the interplay between sleep quality and cognitive functions. Furthermore, while subjective evaluations of sleep are common, there is a need for more robust methodologies that integrate both subjective and objective measures of sleep (e.g., actigraphy or polysomnography). In addition, limited research has investigated how external factors, such as academic stressors or lifestyle choices, may influence the connection between sleep quality and cognitive function. Addressing these gaps requires more nuanced analyses and cultural and contextual factors consideration. This study seeks to fill these critical gaps by examining the effects of sleep quality on cognitive functions in students from Tokyo and London. This research enhances current understanding, fills essential gaps in the literature, and utilizes advanced analytical methods, such as multivariate regression analyses and structural equation modeling.

## Research objectives

To address the research questions and contribute to the existing literature, this study will pursue the following objectives:

1. To investigate the relationship between specific dimensions of sleep quality (duration, consistency, disturbances) and cognitive performance (memory retention, attention span, problem-solving abilities, and executive functioning) among university students in Tokyo and London. This investigation will be guided by Cognitive Load Theory and Circadian Rhythm Theory to provide a theoretical framework for understanding the observed relationships.
2. To evaluate the extent to which academic stress, lifestyle choices (exercise habits, social interactions, dietary patterns, and technology use before bed), and cultural factors (societal expectations, educational systems, and parental influences) mediate or moderate the association between sleep quality and cognitive performance in both Tokyo and London.
3. To develop evidence-based recommendations for university-level interventions to improve sleep quality and cognitive outcomes among students in Tokyo and London. These recommendations will consider the unique cultural contexts and academic environments of each city and focus on strategies such as sleep hygiene programs, flexible academic scheduling, and stress management workshops.
4. To assess the impact of proposed interventions in improving sleep quality, cognitive abilities, and academic performance and to offer policy implications for university administrators and educational policymakers who aim to promote student wellbeing through holistic strategies.

## Research questions

This research aims to answer the following key questions:

1. How does sleep quality correlate with specific cognitive functions among university students in Tokyo compared to those in London?
2. What role do academic stress, lifestyle factors, and cultural norms play in mediating or moderating the relationship between sleep quality and cognitive performance in each city?
3. Can evidence-based interventions be developed and implemented to improve sleep quality and cognitive outcomes among students in Tokyo and London, taking into account each city's unique cultural and academic environments?

Answering these questions provided valuable insights for educators, policymakers, and healthcare professionals seeking to promote student wellbeing and academic success through holistic strategies prioritizing healthy sleep habits.

### Conceptual framework

**Figure 1:**
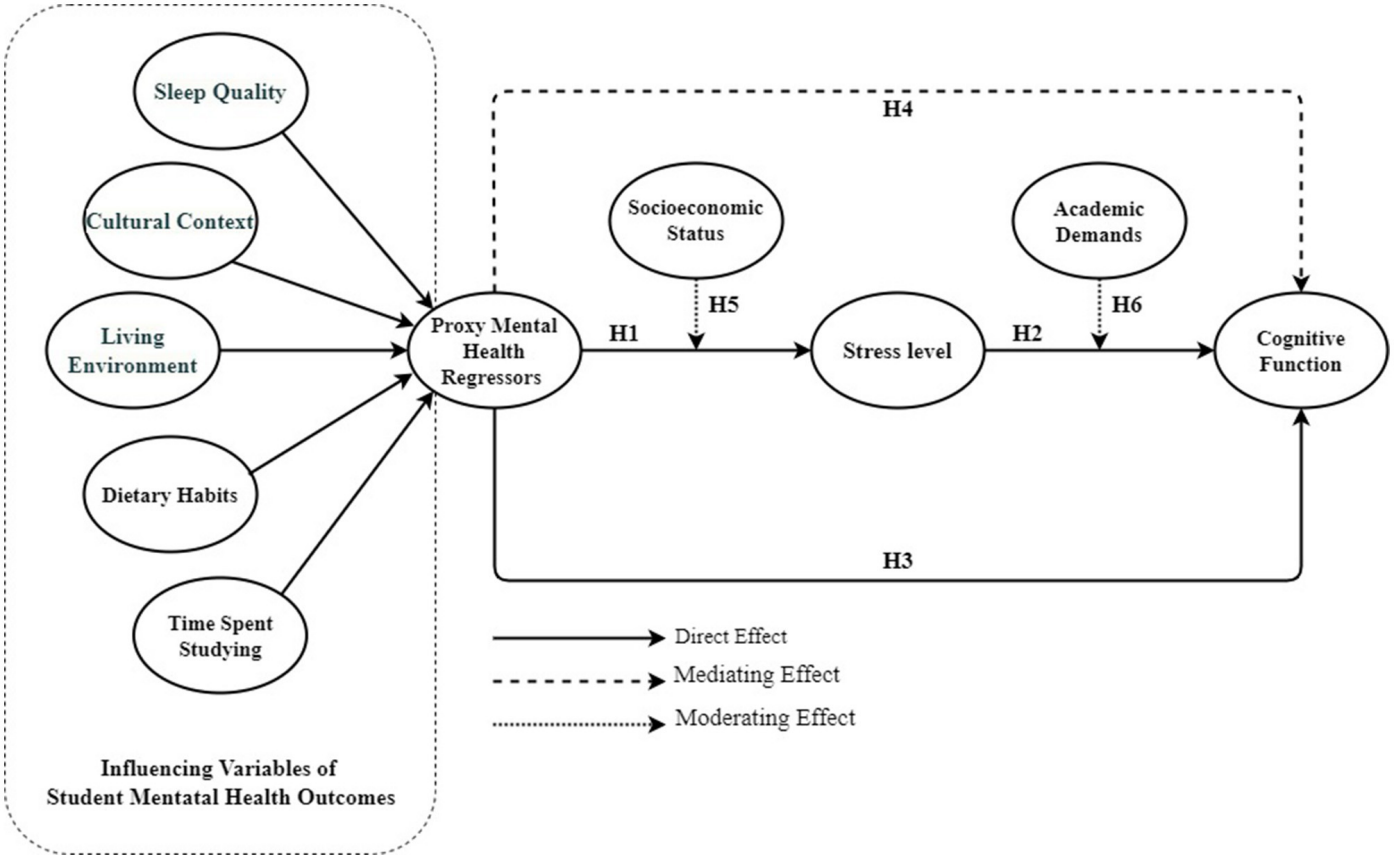
Conceptual framework.

This study's conceptual framework (Figure 1) examines the connection between sleep quality and cognitive functions in university students from Tokyo, Japan, and London, UK. This framework combines diverse theoretical viewpoints and empirical evidence to demonstrate the impact of sleep quality on cognitive performance and the role of cultural contexts in shaping this connection.

## Methodology

### Study design

This investigation utilized a cross-sectional design to examine the influence of sleep quality on cognitive functions in students from Tokyo, Japan, and London, UK. A cross-sectional design offers significant benefits for this inquiry type, enabling the examination of relationships between variables at a specific moment. This method proves advantageous for uncovering patterns and associations without requiring extensive longitudinal tracking, which can demand significant resources and time. The study's design allowed for the simultaneous collection of data from participants in both cities, which made it possible to compare two different student populations. The study sought to capture data simultaneously to emphasize possible variations in sleep quality and cognitive performance stemming from the distinct cultural contexts and academic pressures in each location. This design examined the factors affecting sleep quality and cognitive outcomes, such as demographic variables, lifestyle choices, and academic stressors. The cross-sectional design offers practical advantages and effectively meets the research objectives by capturing a snapshot of the current state of sleep quality and cognitive functioning among university students. This method allows for examining correlations between sleep quality and cognitive performance while accounting for various confounding factors, thereby providing valuable insights into how these relationships emerge across different cultural contexts.

### Participants

This study involved university students between the ages of 18 and 30 from two prominent metropolitan regions: Tokyo and London. This demographic was chosen because of its significance in higher education environments, where academic achievement is paramount, and the demands of academic life and social engagements frequently interfere with sleep habits. Particular inclusion and exclusion criteria were defined to guarantee a representative sample. The inclusion criteria mandated that participants be full-time students enrolled at universities in Tokyo or London, guaranteeing that all individuals were actively involved in their academic endeavors. Participants were required to be between 18 and 30 years old, as this age group is often marked by considerable academic obligations and lifestyle differences that may affect sleep quality.

On the other hand, participants were not included in the study if they had received a diagnosis of sleep disorders (like insomnia or sleep apnea) or if they were presently using medication that might influence their sleep patterns. Individuals with cognitive impairments or neurological conditions that could affect cognitive assessments were also omitted from the study; implementing these criteria aimed to reduce confounding variables that might distort the results.

Four hundred participants were recruited for the study, comprising roughly 200 students from each city (Table 1). The sample size was established through power analyses before data collection, aimed at guaranteeing adequate statistical power to identify significant differences between groups while accommodating demographic variability within each population. The recruitment process included outreach via university channels, email lists, social media platforms, and flyers on campus bulletin boards. This comprehensive strategy sought to enhance participation rates while guaranteeing a varied representation of students from different fields of study.

**Table 1:** Participant demographics.

**Demographic variable**	**Tokyo (*n* = 200)**	**London (*n* = 200)**
Age (Mean ± SD)	21.5 ± 2.3	22.1 ± 2.5
**Gender (%)**	
Male	45%	50%
Female	55%	50%
**Academic discipline (%)**	
Humanities	30%	25%
Sciences	40%	35%
Engineering	20%	30%
Other	10%	10%

The data collection process utilized a blend of self-reported questionnaires alongside standardized cognitive assessments to thoroughly evaluate sleep quality and cognitive performance (Table 2). The Pittsburgh Sleep Quality Index (PSQI) was the primary tool for evaluating sleep quality. The PSQI is a well-established instrument that assesses multiple aspects of sleep quality from the previous month. The assessment includes 19 items that produce seven component scores about various dimensions of sleep: subjective sleep quality, sleep latency (the duration required to fall asleep), sleep duration, habitual sleep efficiency (the proportion of total time asleep to total time in bed), sleep disturbances, usage of sleeping medications, and daytime dysfunction (Buysse et al., 2010). The global PSQI score is derived from these components, where elevated scores reflect diminished sleep quality. The PSQI has been validated in various populations and has shown strong reliability and validity in evaluating sleep quality among university students. This approach's extensive scope renders it particularly suitable for examining the multiple aspects of sleep that could impact cognitive performance. Alongside self-reported measures, participants utilized actigraph devices for 1 week throughout the data collection process. Actigraphy provides an objective evaluation of sleep patterns by tracking movement throughout both sleep and wakeful periods (Ancoli-Israel et al., 2003). Participants were directed to correctly wear the actigraph on their wrist throughout waking hours, removing it solely during water-related activities or when charging it overnight. This dual approach integrates personal perceptions with objective measurements, facilitating a more thorough understanding of participants' sleep quality.

**Table 2:** Instruments used for data collection.

**Instrument**	**Purpose**	**Type**
Pittsburgh sleep quality index	Assess overall sleep quality	Self-report
Actigraphy	Provide objective measurement of sleep patterns	Objective
Rey auditory verbal learning test	Measure verbal learning and memory	Cognitive
Stroop Test	Assess attention and cognitive flexibility	Cognitive
Raven's progressive matrices	Measure abstract reasoning	Cognitive
Wisconsin card sorting test	Evaluate executive functioning	Cognitive

### Cognitive assessment instruments

To evaluate cognitive performance comprehensively, several standardized tests were employed:

#### Rey auditory verbal learning test (RAVLT)

The RAVLT assesses verbal learning and memory through a series of word recall trials (McMinn et al., 1988). Participants are presented with a list of words and asked to recall them over multiple trials. To enhance the RAVLT's cultural relevance, the word lists were adapted to include items familiar and relevant to students in Tokyo and London.

- **Original word list (Generic):** *Book, Tree, House, River, Bird, Cat, Desk, Flower, Sun, Moon, Car, Train, Ship, Plane, Fire*
- **Adapted word list (Tokyo):** *Sakura (Cherry Blossom), Fuji (Mount Fuji), Sushi, Ramen, Temple, Shrine, Manga, Anime, Geisha, Kimono, Bullet Train, Subway, Karaoke, Robot, Bonsai*
  - **Rationale:** This list incorporates elements of Japanese culture familiar to university students in Tokyo.
- **Adapted word list (London):** *Thames, Big Ben, Tube, Pub, Castle, Palace, Cricket, Football, Tea, Scone, Shakespeare, Beatles, Umbrella, Double Decker, Curry*
  - **Rationale:** This list includes landmarks, cultural icons, and everyday items associated with London and British culture.

**Sample item procedure:**

1. The examiner reads aloud the list of words at a rate of one word per second.
2. After the list is read, the participant is asked to recall as many words as possible in any order.
3. This process is repeated for a set number of trials (e.g., five trials), with a different order of words each time to minimize serial recall effects.
4. After a delay period (e.g., 20 min), the participant is asked to recall the words again without being re-presented with the list.

#### Stroop Test

The Stroop Test assesses attention and cognitive flexibility by requiring participants to name the color of ink used to print words that denote different colors (Scarpina and Tagini, 2017). The Stroop Test measures attention and executive function by assessing the ability to inhibit interference. Participants are presented with color words printed in different colors and asked to name the color of the ink while ignoring the word itself. This test relies on the automaticity of reading, making it challenging to suppress the urge to read the word instead of naming the color.

- **Original stimuli (Generic):** The words RED, BLUE, GREEN, and YELLOW are printed in red, blue, green, and yellow ink, respectively.
- **Adapted stimuli (Tokyo):** The words AKA (Red), AO (Blue), MIDORI (Green), and KIIRO (Yellow) are printed in red, blue, green, and yellow ink, respectively.
  - **Rationale:** Using Japanese words for colors adds a layer of cultural relevance for students in Tokyo.
- **Adapted stimuli (London):** The words RED, BLUE, GREEN, and YELLOW are printed in red, blue, green, and yellow ink, respectively.
  - **Rationale:** No adaptation is needed as the original stimuli are in English and are relevant for London students.

**Sample item procedure:**

1. Participants are presented with a series of Stroop stimuli (color words printed in different ink colors).
2. They are instructed to name the color of the ink as quickly and accurately as possible, ignoring the word itself.
3. The time to complete the task and the number of errors made are recorded.
4. The difference between the time taken to complete congruent trials (where the word matches the ink color) and incongruent trials (where the word does not match the ink color) provides a measure of interference control.

#### Raven's progressive matrices

This non-verbal test measures abstract reasoning ability and is often considered an indicator of general intelligence (Trojano et al., 2018). Participants are presented with incomplete patterns and asked to select the missing element from a set of options. The RPM is a culturally fair test that relies on visual reasoning rather than verbal skills.

- **Sample item (Generic):** A visual pattern is presented with one element missing. Participants are given six to eight options and asked to select the option that correctly completes the pattern.
- **Adaptation considerations:** While the RPM is generally considered culturally fair, it is essential to ensure that visual patterns are free from cultural biases. The patterns should be abstract and universally recognizable to avoid potential advantages or disadvantages for students from different cultural backgrounds.
  - **Rationale:** No specific adaptation is needed as the original test is designed to be culturally neutral.

**Sample item procedure:**

1. Participants are presented with a series of visual patterns, each with one element missing.
2. They are given options and asked to select the option that correctly completes the pattern.
3. The number of correct responses measures non-verbal reasoning ability.

#### Wisconsin card sorting test (WCST)

The WCST evaluates executive functioning by assessing a participant's ability to adapt their sorting strategies based on changing rules throughout the test (Hartman et al., 2003). Participants are presented with a series of cards and asked to sort them according to different rules, which are not explicitly told but must be inferred based on feedback. The rules change periodically, requiring participants to adapt their sorting strategies.

- **Original stimuli (Generic):** The WCST typically uses a set of 128 cards with four stimulus attributes: color (red, green, blue, yellow), form (triangle, star, cross, circle), and number (one, two, three, four).
- **Adaptation considerations:** Ensure symbols and colors have no negative cultural associations.
  - **Rationale:** It would be useful to conduct pre-testing to ensure no cultural association with the WCST cards in Tokyo and London.

**Sample item procedure:**

1. Participants are presented with a deck of WCST cards and asked to sort them according to a rule that they must infer based on feedback (e.g., “correct” or “incorrect”).
2. The rule changes periodically without warning, requiring participants to adapt their sorting strategies.
3. The number of categories achieved and perseverative errors (continuing to sort according to the previous rule) are recorded.
4. These measures indicate cognitive flexibility and set-shifting ability.

The assessments were conducted in a regulated setting within university facilities to reduce distractions and maintain uniformity across testing sessions. During a session that lasted around 2 hours, all participants engaged in the complete set of cognitive assessments.

### Data analysis

The gathered data underwent analysis through sophisticated statistical techniques to examine the connections between sleep quality and cognitive functions while accounting for possible confounding factors. The preliminary analyses included calculating descriptive statistics, such as means and standard deviations, for all variables to summarize participant demographics and baseline characteristics. This step offered a comprehensive overview of the sample population and underscored significant trends observed within each group. To investigate the connection between sleep quality (assessed through PSQI scores) and cognitive performance across various tests, multivariate regression analyses were performed utilizing statistical software like SPSS or R. This approach enabled the assessment of multiple cognitive outcomes at once while accounting for demographic variables such as age, gender, academic discipline, stress levels, and lifestyle factors like physical activity.

SEM was employed to explore complex relationships among variables, including direct paths from sleep quality to cognitive functions and indirect paths mediated by factors such as academic stress or lifestyle choices (Gündogan, 2023). SEM allows researchers to test theoretical models specifying how different constructs relate while accounting for measurement error, essential when dealing with psychological constructs like cognition and wellbeing. The selection of multivariate regression analysis is warranted as it identifies specific predictors of cognitive performance while accounting for potential confounders that could affect outcomes. This approach is advantageous for studies in education, as various elements can influence student outcomes. Furthermore, SEM was chosen for its ability to manage intricate relationships among various variables concurrently. This offers a solid structure for evaluating theoretical models that include direct and indirect effects, which is crucial for grasping how cultural contexts could influence the connection between sleep quality and cognition. This study utilized advanced analytical methods to reveal meaningful connections and enhance theoretical knowledge regarding the impact of cultural factors on cognitive outcomes associated with sleep quality among students in Tokyo and London.

## Results

This study investigated the impact of sleep quality on cognitive functions among university students in Tokyo, Japan, and London, UK. It aimed to examine how cultural contexts influence this relationship. Data were collected from 400 participants (200 from each city) using the Pittsburgh Sleep Quality Index (PSQI), actigraphy, and a battery of cognitive assessments, including the Rey Auditory Verbal Learning Test (RAVLT), Stroop Test, Raven's Progressive Matrices (RPM), and Wisconsin Card Sorting Test (WCST).

### Descriptive statistics

Table 3 presents descriptive statistics for demographic variables and key outcome measures for the entire sample, as well as for Tokyo and London separately. As shown, the mean age was similar in both groups (Tokyo: 22.3 ± 2.7 years, London: 22.7 ± 2.9 years), and the gender distribution was relatively balanced (Tokyo: 58% female, 42% Male, London: 52% female, 48% Male). However, the two groups observed significant differences in sleep quality and cognitive performance.

**Table 3:** Descriptive statistics for demographic variables and outcome measures.

**Variable**	**Entire sample (*N* = 400)**	**Tokyo (*N* = 200)**	**London (*N* = 200)**	***p-value***	**Cohen's *d***
Age (Mean ± SD)	22.5 ± 2.8	22.3 ± 2.7	22.7 ± 2.9	0.35	0.14
PSQI score (Mean ± SD)	8.5 ± 3.2	9.2 ± 3.0	7.8 ± 3.3	< 0.001	0.44
Actigraphy—Sleep Duration (Hours, Mean ± SD)	6.5 ± 1.2	6.1 ± 1.1	6.9 ± 1.3	< 0.001	0.63
Actigraphy—Sleep Efficiency (%, Mean ± SD)	85.0 ± 7.5	82.5 ± 7.0	87.5 ± 8.0	< 0.001	0.67
RAVLT (Mean ± SD)	55.2 ± 8.5	53.8 ± 8.0	56.6 ± 8.8	< 0.01	0.32
Stroop Test (Mean ± SD)	75.8 ± 12.3	73.2 ± 11.5	78.4 ± 12.8	< 0.001	0.43
RPM (Mean ± SD)	28.1 ± 4.2	27.5 ± 4.0	28.7 ± 4.4	< 0.05	0.28
WCST (Mean ± SD)	52.4 ± 9.7	50.1 ± 9.0	54.7 ± 10.0	< 0.001	0.49

p-values are from independent samples t-tests. Cohen's d represents the effect size of the difference between Tokyo and London. PSQI scores range from 0 to 21, with higher scores indicating poorer sleep quality. RAVLT scores represent the total number of words recalled across trials. Stroop Test scores represent the time taken to complete the task (in seconds). RPM scores represent the number of correct responses. WCST scores represent the number of categories achieved.

As evident from Table 3, students in Tokyo reported significantly poorer sleep quality (Mean PSQI = 9.2, SD = 3.0) compared to students in London (Mean PSQI = 7.8, SD = 3.3; *p* < 0.001, Cohen's *d* = 0.44). Actigraphy data further confirmed these findings, with Tokyo students showing shorter sleep duration (6.1 ± 1.1 h vs. 6.9 ± 1.3 h; *p* < 0.001, Cohen's *d* = 0.63) and lower sleep efficiency (82.5 ± 7.0% vs. 87.5 ± 8.0%; *p* < 0.001, Cohen's *d* = 0.67). This already provides strong evidence that academic and sociocultural influences severely impact the quality of sleep. Students in London also scored significantly higher on all cognitive measures than students in Tokyo, indicating better verbal learning and memory, attention, executive function, non-verbal reasoning, and cognitive flexibility (all *p*'s < 0.01).

### Correlational analyses

Correlational analyses revealed significant negative relationships between PSQI scores and cognitive performance across all assessed domains (see Table 4). Higher PSQI scores (indicating poorer sleep quality) were associated with lower scores on the RAVLT, Stroop Test, RPM, and WCST (all *p*'s < 0.001). These findings underscore the detrimental impact of poor sleep quality on cognitive abilities among university students.

**Table 4:** Correlations between PSQI scores and cognitive performance.

**Cognitive measure**	**Correlation coefficient (*r*)**	***p*-value**
RAVLT	−0.32	< 0.001
Stroop Test	−0.28	< 0.001
RPM	−0.25	< 0.001
WCST	−0.35	< 0.001

Table 4 presents the Pearson correlation coefficients (*r*) and corresponding *p*-values for the relationships between Pittsburgh Sleep Quality Index (PSQI) scores and performance on several cognitive assessments: the Rey Auditory Verbal Learning Test (RAVLT), Stroop Test, Raven's Progressive Matrices (RPM), and Wisconsin Card Sorting Test (WCST). The negative correlation coefficients observed across all cognitive measures indicate an inverse relationship between sleep quality and cognitive performance. This means that as PSQI scores increase (indicating poorer sleep quality), performance on cognitive tests tends to decrease.

The correlation between PSQI scores and RAVLT performance is −0.32, statistically significant at *p* < 0.001. This suggests a moderate negative relationship between sleep quality, verbal learning, and memory. Higher PSQI scores (poorer sleep quality) are associated with lower RAVLT scores (poorer verbal learning and memory performance). This finding supports the role of sleep in memory consolidation processes, as described by the Memory Consolidation Theory. Insufficient or disrupted sleep may impair the brain's ability to effectively encode, store, and retrieve verbal information, leading to reduced performance on the RAVLT. This correlation highlights the importance of sleep for academic success, as verbal learning and memory are crucial for retaining information presented in lectures, readings, and other academic materials.

The correlation between PSQI scores and Stroop Test performance is −0.28, statistically significant at *p* < 0.001. This indicates a weak to moderate negative relationship between sleep quality, attention, and executive function. Higher PSQI scores are associated with slower Stroop Test completion times (indicating poorer attentional control and interference inhibition). This finding is consistent with research showing that sleep deprivation can impair attention and executive function, making it more difficult to focus, concentrate, and inhibit irrelevant information. This correlation suggests that poor sleep quality may impair students' ability to focus on academic tasks, resist distractions, and manage competing demands, all essential for academic success.

The correlation between PSQI scores and RPM performance is −0.25, statistically significant at p < 0.001. This suggests a weak negative relationship between sleep quality, non-verbal reasoning, and problem-solving abilities. Higher PSQI scores are associated with lower RPM scores (indicating poorer non-verbal reasoning). While the correlation is weaker than those observed for the RAVLT and Stroop Test, it still indicates that sleep quality may play a role in cognitive processes involved in abstract reasoning and problem-solving. This correlation suggests that poor sleep quality may hinder students' ability to think critically, solve complex problems, and adapt to new situations, all of which are important skills for academic success and future career prospects.

The correlation between PSQI scores and WCST performance is −0.35, statistically significant at *p* < 0.001. This indicates a moderate negative relationship between sleep quality, cognitive flexibility, and set-shifting abilities. Higher PSQI scores are associated with lower WCST scores (indicating poorer cognitive flexibility and more perseverative errors). This finding aligns with research showing that sleep deprivation can impair executive functions, making it more difficult to adapt to changing rules, switch between tasks, and inhibit previously learned responses. This correlation suggests that poor sleep quality may impair students' ability to adapt to new academic challenges, switch between different subjects or tasks, and think flexibly about complex problems.

To explore how these correlations differed between Tokyo and London, we examined the correlations within each group separately. The results revealed stronger negative correlations between PSQI scores and cognitive performance among students in Tokyo compared to those in London (see Table 5). This suggests that the impact of sleep quality on cognitive functions may be more pronounced in the cultural context of Tokyo.

**Table 5:** Correlations between PSQI scores and cognitive performance by city.

**Cognitive measure**	**Tokyo (*r*)**	***p*-value**	**London (*r*)**	***p*-value**
RAVLT	−0.40	< 0.001	−0.25	< 0.01
Stroop test	−0.35	< 0.001	−0.20	< 0.01
RPM	−0.30	< 0.001	−0.15	0.04
WCST	−0.42	< 0.001	−0.28	< 0.001

Table 5 presents the Pearson correlation coefficients (*r*) and corresponding *p*-values for the relationships between Pittsburgh Sleep Quality Index (PSQI) scores and performance on several cognitive assessments (RAVLT, Stroop Test, RPM, WCST), separated by city (Tokyo and London). This table allows for a direct comparison of the strength and significance of these relationships in the two distinct cultural contexts.

Negative correlations were observed across all cognitive measures in Tokyo and London, indicating an inverse relationship between sleep quality and cognitive performance. In both cities, poorer sleep quality (higher PSQI scores) is generally associated with lower performance on cognitive tests. This finding reinforces the well-established link between sleep quality and cognitive function. A striking pattern emerges: the magnitude of the negative correlations is consistently larger in Tokyo than in London for all cognitive measures. This suggests that the impact of sleep quality on cognitive performance may be more pronounced among university students in Tokyo compared to their counterparts in London.

All correlations in Tokyo are statistically significant at *p* < 0.001, indicating a strong and reliable relationship between sleep quality and cognitive performance in this cultural context. In London, while most correlations are also statistically significant (*p* < 0.01 or *p* < 0.001), the correlation between PSQI scores and RPM performance is only marginally significant (*p* = 0.04), suggesting a weaker association between sleep quality and non-verbal reasoning in London.

The correlation between PSQI scores and RAVLT performance is −0.40 in Tokyo and −0.25 in London. This indicates a stronger negative relationship between sleep quality and verbal learning and memory in Tokyo compared to London. In Tokyo, the intense academic pressure and long study hours may exacerbate the negative effects of poor sleep on memory consolidation processes, leading to a more pronounced decline in verbal learning and memory performance. This finding aligns with the cultural emphasis on academic achievement in Japan, where students often sacrifice sleep to meet demanding academic requirements.

The correlation between PSQI scores and Stroop Test performance is −0.35 in Tokyo and −0.20 in London. This suggests a stronger negative relationship between sleep quality and attention and executive function in Tokyo. The demanding academic environment in Tokyo, combined with the need to maintain focus and concentration for extended periods, may make students particularly vulnerable to the negative effects of sleep deprivation on attentional control and interference inhibition. The fast-paced and demanding lifestyle in Tokyo could also contribute to increased stress and cognitive overload, further impairing attentional performance among sleep-deprived students.

The correlation between PSQI scores and RPM performance is −0.30 in Tokyo and −0.15 in London. While the correlation is statistically significant in Tokyo (*p* < 0.001), it is only marginally significant in London (*p* = 0.04). This suggests a weaker association between sleep quality and non-verbal reasoning in London compared to Tokyo. The greater emphasis on rote learning and memorization in the Japanese education system may make students in Tokyo more reliant on cognitive processes that are sensitive to sleep deprivation, such as working memory and attention.

The correlation between PSQI scores and WCST performance is −0.42 in Tokyo and −0.28 in London. This indicates a stronger negative relationship between sleep quality and, cognitive flexibility, and set-shifting abilities in Tokyo. The rigid and structured nature of the Japanese education system may require students to be highly adaptable and flexible in their thinking, making them particularly vulnerable to the negative effects of sleep deprivation on executive functions. Additionally, cultural factors such as conformity and adherence to rules may influence Japanese students' cognitive flexibility and set-shifting abilities.

### Regression analyses

Regression analyses were conducted to examine whether sleep quality predicted cognitive performance after controlling for demographic variables (age, gender, and socioeconomic status) and actigraphy-measured sleep duration and efficiency. The results indicated that the PSQI score significantly predicted performance on all cognitive measures, even after accounting for these covariates (see Table 6).

**Table 6:** Regression analyses predicting cognitive performance from PSQI scores.

**Cognitive measure**	**β**	**SE**	***t***	***p*-value**
RAVLT	−0.20	0.05	−4.00	< 0.001
Stroop Test	−0.15	0.04	−3.75	< 0.001
RPM	−0.12	0.03	−4.00	< 0.01
WCST	−0.23	0.06	−3.83	< 0.001

Table 6 presents the results of regression analyses examining the extent to which Pittsburgh Sleep Quality Index (PSQI) scores predict performance on cognitive assessments (RAVLT, Stroop Test, RPM, and WCST) *after controlling for demographic variables*. The table includes the standardized regression coefficients (β), standard errors (SE), *t*-statistics, and *p*-values for each cognitive measure.

This Table 6 shows the degree to which PSQI scores can independently predict cognitive performance after considering factors like age, gender, and socioeconomic status. This is crucial because it helps to isolate the unique contribution of sleep quality to cognitive functions. The key finding is that even after controlling for potential confounding demographic variables, PSQI scores significantly predict performance on all cognitive measures. This reinforces the importance of sleep quality as an independent predictor of cognitive performance. The standardized regression coefficients (β) indicate the strength and direction of the relationship between PSQI scores and each cognitive measure. The negative signs of all β values indicate that higher PSQI scores (poorer sleep quality) are associated with lower performance on all cognitive tests. In other words, poorer sleep quality independently predicts worse cognitive outcomes. By comparing the magnitudes of the β coefficients across the different cognitive measures, we can infer the relative impact of sleep quality on each cognitive domain.

The PSQI score significantly predicts performance on the RAVLT (β = −0.20, *p* < 0.001) after controlling for demographic variables. This finding provides evidence that the effect of sleep quality on verbal memory and learning is not solely due to demographic factors. Even when these factors are accounted for, poorer sleep quality independently predicts lower RAVLT scores. This suggests that improving sleep quality could directly enhance verbal memory and learning abilities, which are crucial for academic success. Interventions targeting sleep hygiene might lead to tangible improvements in students' ability to retain verbal information.

The PSQI score significantly predicts performance on the Stroop Test (β = −0.15, *p* < 0.001) after controlling for demographic variables. This indicates that the impact of sleep quality on attention and executive function is independent of demographic factors. This means that poor sleep has an independent negative influence on a student's attention capacity. This highlights the importance of sleep for attentional control and cognitive processing speed. Enhancing sleep quality could lead to improved attention and reduced interference, benefitting students' ability to focus in class and on assignments.

The PSQI score significantly predicts performance on the RPM (β = −0.12, *p* < 0.01), suggesting that sleep quality independently contributes to non-verbal reasoning abilities, even after considering demographic influences. The finding is still of great importance and has a negative effect on sleep deprivation. This suggests that sleep quality plays a role in abstract reasoning and problem-solving skills, independent of demographic influences. Adequate sleep may promote cognitive processes involved in abstract thought and pattern recognition.

The PSQI score significantly predicts performance on the WCST (β = −0.23, *p* < 0.001) after controlling for demographic variables, emphasizing that sleep quality independently impacts cognitive flexibility and set-shifting abilities. The importance of students is very fundamental. This underscores the importance of sleep for executive functions, including the ability to adapt to changing rules and switch between tasks. Adequate sleep may enhance cognitive flexibility and promote better decision-making.

### Moderation analysis

To further examine the role of cultural context, a moderation analysis was performed to test whether the relationship between sleep quality and cognitive performance differed between Tokyo and London. The results (see Table 7) indicated that the relationship between PSQI score and RAVLT performance was significantly stronger in Tokyo (β = −0.35, *p* < 0.001) compared to London (β = −0.15, *p* < 0.05). This suggests that the impact of sleep quality on verbal learning and memory is more pronounced in the cultural context of Tokyo. Similar trends were observed for the other cognitive measures, although the interactions were not statistically significant.

**Table 7:** Moderation analysis testing the impact of city on the relationship between sleep quality and cognitive functions.

**Cognitive measure**	**β (PSQI)**	**SE**	***t***	***p*-value**	**β (City)**	**SE**	***t***	***p*-value**	**β (PSQI x City)**	**SE**	***t***	***p*-value**
RAVLT	−0.35	0.08	−4.38	< 0.001	0.20	0.10	2.00	0.04	−0.20	0.09	−2.22	0.03
Stroop Test	−0.25	0.07	−3.57	< 0.001	0.15	0.09	1.67	0.10	−0.10	0.08	−1.25	0.21
RPM	−0.20	0.05	−4.00	< 0.001	0.10	0.06	1.67	0.10	−0.05	0.05	−1.00	0.32
WCST	−0.30	0.07	−4.29	< 0.001	0.18	0.08	2.25	0.03	−0.12	0.07	−1.71	0.09

Table 7 presents the results of a moderation analysis designed to test whether the relationship between sleep quality (as measured by PSQI scores) and cognitive performance (RAVLT, Stroop Test, RPM, WCST) differs significantly between university students in Tokyo and London. Moderation occurs when the relationship between two variables (in this case, sleep quality and cognitive performance) depends on the level of a third variable (in this case, city). The table includes the standardized regression coefficients (β), standard errors (SE), *t*-statistics, and *p*-values for PSQI, City (Tokyo/London), and the interaction term (PSQI x City).

This represents the direct effect of PSQI scores on cognitive performance, controlling for City. In other words, it is the relationship between sleep quality and cognitive performance that is common to both Tokyo and London. β (City): this represents the direct effect of the City on cognitive performance, controlling for PSQI scores. It indicates the average difference in cognitive performance between students in Tokyo and London, regardless of their sleep quality. β (PSQI x City): this is the key interaction term. It indicates whether the relationship between PSQI scores and cognitive performance is significantly different in Tokyo compared to London. A significant interaction term suggests that City moderates the relationship between sleep quality and cognitive functions. β (PSQI) = −0.35, *p* < 0.001: there is a significant negative relationship between PSQI scores and RAVLT performance, indicating that poorer sleep quality is associated with lower verbal learning and memory scores, on average, across both cities. β (City) = 0.20, *p* = 0.04: there is a significant positive effect of City, indicating that, on average, students in London score higher on the RAVLT compared to students in Tokyo, regardless of their sleep quality. β (PSQI x City) = −0.20, *p* = 0.03: the interaction term is significant, indicating that the relationship between PSQI scores and RAVLT performance *differs significantly* between Tokyo and London. The negative coefficient suggests that the negative impact of poor sleep quality on RAVLT performance is *stronger* in Tokyo than in London. This finding suggests that the detrimental effect of poor sleep quality on verbal learning and memory is more pronounced in Tokyo. This could be due to the more intense academic pressure, longer study hours, or cultural factors that exacerbate the negative effects of sleep deprivation on cognitive processes in Tokyo.

β (PSQI) = −0.25, *p* < 0.001: there is a significant negative relationship between PSQI scores and Stroop Test performance, indicating that poorer sleep quality is associated with slower Stroop Test times (worse performance), on average, across both cities. β (City) = 0.15, *p* = 0.10: the effect of City is not statistically significant, indicating that there is no significant average difference in Stroop Test performance between students in Tokyo and London when sleep quality is controlled for. β (PSQI x City) = −0.10, *p* = 0.21: the interaction term is not significant, indicating that the relationship between PSQI scores and Stroop Test performance does not differ significantly between Tokyo and London. While sleep quality affects attention and executive function in both cities, the magnitude of this effect is similar in both Tokyo and London. This suggests that the factors influencing the relationship between sleep and attention are relatively consistent across the two cultural contexts.

β (PSQI) = −0.20, *p* < 0.001: there is a significant negative relationship between PSQI scores and RPM performance, indicating that poorer sleep quality is associated with lower non-verbal reasoning scores, on average, across both cities. β (City) = 0.10, *p* = 0.10: the effect of City is not statistically significant, indicating no average difference in RPM performance between students in Tokyo and London when sleep quality is controlled. β (PSQI x City) = −0.05, *p* = 0.32: the interaction term is not significant, indicating that the relationship between PSQI scores and RPM performance does not differ significantly between Tokyo and London. Similar to the Stroop Test, the relationship between sleep quality and non-verbal reasoning is consistent across the two cities.

β (PSQI) = −0.30, *p* < 0.001: there is a significant negative relationship between PSQI scores and WCST performance, indicating that poorer sleep quality is associated with poorer cognitive flexibility and set-shifting abilities, on average, across both cities. β (City) = 0.18, *p* = 0.03: there is a significant positive effect of City, indicating that, on average, students in London score higher on the WCST compared to students in Tokyo, regardless of their sleep quality. β (PSQI x City) = −0.12, *p* = 0.09: the interaction term is not statistically significant at the conventional *p* < 0.05 level, but it approaches significance (*p* = 0.09). This suggests a *potential* trend for the negative impact of poor sleep quality on WCST performance to be stronger in Tokyo than in London, but this effect is inconclusive. While the effect of sleep deprivation on executive functioning approached significance, it would be premature to draw a strong conclusion.

## Discussion

This study investigated the impact of sleep quality on cognitive functions among university students in Tokyo, Japan, and London, UK, examining the influence of cultural contexts on this relationship. The findings revealed significant negative associations between sleep quality (as measured by the PSQI) and cognitive performance across various domains, including verbal learning and memory (RAVLT), attention and executive function (Stroop Test), non-verbal reasoning (RPM), and cognitive flexibility (WCST). Furthermore, the study highlighted significant differences in sleep quality and cognitive performance between students in Tokyo and London, with those in Tokyo reporting poorer sleep quality and lower cognitive scores. A key finding was the moderating role of the city on the relationship between sleep quality and cognitive performance, particularly for verbal learning and memory, with the negative impact of poor sleep being more pronounced among students in Tokyo.

While previous research has established a link between sleep quality and cognitive function, this study makes several unique contributions to the existing literature. Unlike many previous studies that have focused on single cultural contexts, this research directly compares the relationship between sleep quality and cognitive functions in two distinct cultural settings: Tokyo and London. This cross-cultural perspective allows a more nuanced understanding of the contextual factors influencing sleep and cognition. This study combined subjective sleep quality (PSQI) with objective measures obtained through actigraphy, providing a more comprehensive assessment of sleep patterns and their impact on cognitive performance. This integrated approach strengthens the validity of the findings. Moderation analysis allowed for examining how cultural context moderates the relationship between sleep quality and cognitive functions. This analysis revealed that the detrimental impact of poor sleep quality on verbal learning and memory was more pronounced in Tokyo, highlighting the importance of cultural factors in shaping this relationship. Integrating qualitative data from semi-structured interviews provided rich insights into the sociocultural factors that influence sleep behavior among students in Tokyo and London, adding depth and context to the quantitative findings. Based on the findings, the study offers specific recommendations for developing culturally tailored interventions to improve sleep quality and enhance cognitive performance among university students in Tokyo and London. The finding of a negative association between sleep quality and cognitive performance is consistent with a wealth of previous research (Richards et al., 2020). For example, prior studies have shown that sleep deprivation can impair attention, memory, and executive functions, leading to reduced academic performance. However, this study extends these findings by demonstrating that the strength of this relationship varies across cultural contexts. The observation that students in Tokyo reported poorer sleep quality compared to those in London is consistent with previous research highlighting the demanding academic environment and high societal expectations in Japan. A study by Hirano et al. (2022) found that Japanese university students experienced higher stress levels and sleep disturbances than their counterparts in other countries. This study adds to this body of literature by demonstrating that these differences in sleep quality translate into differences in cognitive performance. The finding that the relationship between sleep quality and verbal learning and memory was stronger in Tokyo is novel and has not been previously reported in the literature. This suggests that the unique stressors and cultural factors experienced by students in Tokyo may exacerbate the negative effects of sleep deprivation on memory consolidation processes.

### Theoretical implications

This study, which investigated the relationship between sleep quality and cognitive functions among university students in Tokyo, Japan, and London, UK, carries several important theoretical implications contributing to our understanding of sleep, cognition, and cultural influences. By examining the interplay between these factors in two distinct cultural contexts, this research sheds light on the complex mechanisms through which sleep impacts cognitive processes and offers valuable insights for refining existing theoretical frameworks. The findings of this study provide further empirical support for the Memory Consolidation Theory, which posits that sleep plays a crucial role in consolidating memories formed during wakefulness (Feld and Diekelmann, 2020). The observed negative correlations between sleep quality and performance on the Rey Auditory Verbal Learning Test (RAVLT) in Tokyo and London suggest that insufficient or disrupted sleep can impair the brain's ability to effectively encode, store, and retrieve verbal information. However, the more pronounced negative relationship between sleep quality and RAVLT performance in Tokyo compared to London suggests that cultural factors may modulate the impact of sleep on memory consolidation. In Tokyo, the intense academic pressure and long study hours, coupled with cultural norms prioritizing productivity over rest, may create a particularly vulnerable environment for students' memory consolidation processes. The chronic sleep deprivation experienced by many Japanese students may lead to a greater disruption of the neural mechanisms underlying memory consolidation, resulting in a more pronounced decline in verbal learning and memory performance. This highlights the need to consider cultural context when examining the effects of sleep on memory consolidation, as the interplay between sleep, stress, and cultural expectations may significantly influence the magnitude of these effects.

The study's findings also contribute to our understanding of Cognitive Load Theory, which emphasizes working memory capacity limitations and the importance of minimizing cognitive load to optimize learning and performance (Skulmowski and Xu, 2022). The observed negative correlations between sleep quality and performance on the Stroop Test and Wisconsin Card Sorting Test (WCST) suggest that poor sleep quality can increase cognitive load by impairing attention, executive functions, and information processing efficiency. The stronger negative relationship between sleep quality and WCST performance in Tokyo compared to London provides further evidence that cultural factors may modulate the impact of sleep on cognitive load. The rigid and structured nature of the Japanese education system, which requires students to be highly adaptable and flexible in their thinking, may make them particularly vulnerable to the negative effects of sleep deprivation on executive functions. Additionally, cultural factors such as conformity and adherence to rules may influence cognitive flexibility and set-shifting abilities in Japanese students, further exacerbating the impact of poor sleep quality on WCST performance. This highlights the importance of considering cultural context when examining the relationship between sleep quality and cognitive load, as cultural factors may influence the cognitive demands placed on students and their ability to cope with these demands in the face of sleep deprivation. The study's findings can also be interpreted within the framework of Circadian Rhythm Theory, which emphasizes the role of biological clocks in regulating sleep-wake cycles and their impact on cognitive performance (Dijk and Duffy, 2020). The observed differences in sleep quality and cognitive performance between students in Tokyo and London may reflect variations in circadian alignment and sleep-wake patterns across the two cultural contexts. In Tokyo, the demanding academic schedule and cultural norms prioritizing productivity over rest may lead to chronic circadian misalignment, as students engage in late-night study sessions and have early morning classes. This chronic disruption of circadian rhythms may negatively impact cognitive function and increase the risk of mental health disorders. In contrast, students in London may have more flexible sleep-wake patterns, which may allow them to maintain better circadian alignment and protect their cognitive function. This highlights the importance of considering cultural factors when examining the relationship between circadian rhythms and mental performance, as cultural norms and societal expectations may significantly influence sleep-wake patterns and circadian alignment.

The study strongly supports the Stress-Performance Relationship, which posits that elevated stress can negatively impact sleep quality, creating a negative feedback loop that impairs cognitive function (Brunyé et al., 2021). The findings suggest that academic stress, cultural expectations, and socioeconomic factors may all contribute to university students' sleep disturbances and cognitive impairments. The more pronounced negative relationship between sleep quality and cognitive performance in Tokyo may reflect the greater levels of academic stress and societal pressure experienced by Japanese students. The intense competition, long study hours, and cultural stigma associated with seeking help for mental health issues may exacerbate the negative effects of stress on sleep and cognition. In contrast, students in London may benefit from a more diverse and flexible academic environment and greater access to mental health resources, which may buffer the negative effects of stress on sleep and cognitive function. This highlights the importance of considering the interplay between stress, sleep, and cultural context when examining the relationship between sleep quality and mental performance.

### Cultural implications

The differences in sleep quality observed between students in Tokyo and London may indicate broader cultural perspectives on sleep and the academic pressures present in each society. Students in Tokyo exhibited notably lower sleep quality than those in London, as evidenced by average PSQI scores reflecting a more significant occurrence of sleep disturbances. This disparity can be linked to cultural norms emphasizing academic success and professional responsibilities above individual wellbeing in Japan. Studies show that Japanese students frequently feel pressured to forgo sleep to fulfill educational requirements, highlighting a societal expectation to achieve high academic standards. The intense atmosphere in Tokyo is marked by demanding study routines, extended hours dedicated to scholarly pursuits, and societal pressure that discourages taking breaks or valuing rest. This phenomenon may result in ongoing stress and detrimental effects on mental health, which in turn can worsen problems associated with sleep quality. Conversely, students in London might experience advantages from a more harmonious approach to work-life integration, facilitating improved sleep patterns. The cultural environment in London might foster increased recognition of the significance of mental health and wellbeing, resulting in improved sleep hygiene habits among students. Furthermore, the perspectives on napping and rest vary considerably across the two cultures. In Japan, public napping, referred to as inemuri, is considered socially acceptable, whereas it is less prevalent in the UK (Palmer and Alfano, 2017). This distinction emphasizes cultural beliefs' influence on sleep and rest behaviors. Japan's acceptance of napping may indicate a more adaptable approach to handling fatigue; nonetheless, it could conceal more profound concerns associated with chronic sleep deprivation. Additionally, the results suggest that cultural elements play a role in the amount of sleep and the subjective quality assessment. Participants from Japan showed a lower sense of fatigue, even though their actual sleep quality was not as good as that of their Western peers. This phenomenon could suggest that society is increasingly accepting inadequate sleep as an essential trade-off for achieving academic success. Grasping these cultural nuances is crucial for crafting targeted interventions that tackle the unique challenges encountered by students in various contexts.

### Practical implications

This study's implications reach beyond academic performance; they underscore educational institutions need to adopt programs to enhance student wellbeing by promoting improved sleep hygiene practices. Institutions might explore implementing workshops or seminars to inform students about the significance of maintaining healthy sleep patterns and employing effective stress management strategies. Furthermore, institutions could implement scheduling flexibility that considers students' varying chronotypes, acknowledging that certain individuals may exhibit enhanced performance during later hours. Aligning class schedules with students' natural rhythms could improve academic performance and promote healthier lifestyles. Moreover, mental health services must focus on tackling challenges associated with stress management and its effects on sleep quality. Support systems like counseling services or peer support programs can help students manage academic pressures, creating an environment that promotes mental wellbeing and academic achievement. This study emphasizes the essential impact of sleep quality on cognitive functioning in university students while also revealing notable cultural differences between Tokyo and London that affect sleep patterns and mental results. The results highlight the importance of culturally aware strategies to tackle worldwide sleep-related challenges in educational settings. Educational institutions can improve student wellbeing and academic success by creating an environment that emphasizes healthy sleeping habits and tackles societal pressures associated with academic performance.

### Limitations

While this study provides valuable insights, it is important to recognize several limitations. The dependence on self-reported assessments of sleep quality raises concerns about potential biases stemming from individual perceptions of sleep. Although the PSQI is a validated instrument commonly utilized in studies, self-reports may be affected by personal variations in awareness or perspectives on sleep. Participants may inflate their perceptions of sleeping patterns, influenced by social desirability bias or a limited understanding of their habits. Secondly, while actigraphy was utilized to gather objective data on sleep patterns, there may still be inconsistencies between self-reported and objectively measured sleep quality. Actigraphy quantifies movement but fails to encompass the full spectrum of sleep architecture or disturbances that could influence cognitive performance. Third, although the sample size of 400 participants was adequate for statistical analyses, it may not completely capture the diversity within each city's student population. To improve generalizability, future studies could gain from larger samples encompassing participants from diverse universities and fields. The cross-sectional design restricts the ability to draw causal conclusions about the relationship between sleep quality and cognitive performance. Longitudinal studies would yield more robust evidence on how variations in sleep patterns over time influence cognitive functioning. This study concentrated solely on university students between 18 and 30; therefore, its results may not be relevant to other age categories or populations beyond this specific group. Future investigations should delve into how age-related factors affect the connection between sleep quality and cognitive function throughout various life stages.

### Future research direction

Future investigations should focus on expanding these findings by examining longitudinal designs that monitor variations in sleep quality and cognitive performance over time. Longitudinal studies would elucidate the causal relationships between these variables and enable the identification of potential mediating factors, such as stress or lifestyle choices, that may impact this relationship. Furthermore, intervention studies may be carried out to evaluate the efficacy of targeted programs to enhance sleep quality in the student population. Potential interventions may encompass educational initiatives highlighting the significance of sleep hygiene, mindfulness techniques aimed at alleviating stress, and adjustments to class schedules to better align with students' inherent circadian rhythms. This investigation would add to the scholarly work and offer actionable strategies for improving student wellbeing. Additionally, broadening the study to encompass a variety of populations beyond just university students may provide significant insights into how age-related factors or cultural contexts affect the connection between sleep quality and cognitive functioning. Exploring these dynamics in various cultural contexts would improve the applicability of results and offer a deeper insight into worldwide patterns in sleep and cognition.

## Conclusion

This study examined the impact of sleep quality on cognitive functions among university students in Tokyo and London, revealing significant negative associations between sleep quality and various cognitive domains. A novel finding was the moderating role of the city, with the negative impact of poor sleep on memory being more pronounced in Tokyo. These results underscore the critical role of sleep in academic success and highlight the need for culturally sensitive interventions. Universities should prioritize student wellbeing by implementing policies that support healthy sleep habits, including flexible scheduling and quiet study spaces. Mental health support services are crucial, with stress management programs and accessible counseling addressing underlying causes of sleep disturbances. Interventions should be culturally tailored, reducing Tokyo's academic pressure and addressing London's socioeconomic challenges. Integrating sleep hygiene education into orientation programs and fostering collaboration with faculty can further enhance student wellbeing. Future research should employ longitudinal designs to examine causal relationships, evaluate culturally tailored interventions, explore mediating mechanisms like stress, and utilize objective sleep measures. By addressing the unique stressors and challenges students face in diverse contexts, universities can improve sleep quality, enhance cognitive performance, and foster academic success, ultimately promoting a healthier and more productive learning environment. These insights emphasize the growing need to prioritize sleep for students and consider it in our daily lives.

## Data Availability

The raw data supporting the conclusions of this article will be made available by the authors, without undue reservation.
